# Study on the *P-S-N* Curve of Sucker Rod Based on Three-Parameter Weibull Distribution

**DOI:** 10.3390/ma15020560

**Published:** 2022-01-12

**Authors:** Wenbin Cai, Wen Li, Jinze Xu

**Affiliations:** 1 College of Petroleum Engineering, Xi’an Shiyou University, Xi’an 710065, China; caiwenbin@xsyu.edu.cn; 2 Department of Chemical and Petroleum Engineering, University of Calgary, Calgary, AB T2N 1N4, Canada; jinzxu@ucalgary.ca

**Keywords:** ultra-high strength sucker rod, fatigue life, Weibull distribution, *P*-*S*-*N* curve

## Abstract

During the oil production process, sucker rods are subjected to cyclic alternating load. After a certain number of cycles, a sucker rod can experience fatigue failure. The number of cycles is called fatigue life (*N*), and the accurate relationship between maximum stress (*S*) and fatigue life (*N*) under a certain reliability (*P*), namely the *P*-*S*-*N* curve, is an important basis for the reliability analysis and fatigue life prediction of sucker rods. The Basquin model, based on log-normal distribution, is widely used for fitting the *P*-*S*-*N* curves of sucker rods. Due to the limitation of this model, it is difficult to extrapolate the conclusion obtained from a finite fatigue region to the high-cycle or ultra-high-cycle fatigue region, which makes it impossible to estimate the fatigue limit of the sucker rod. Compared to the log-normal distribution, Weibull distribution causes the sucker rod to have a minimum safety life, namely the safety life at 100% survival rate, which complies with the fatigue characteristics of the sucker rod and is more in line with the actual situation. In this study, the fatigue data for ultra-high-strength HL and HY grade sucker rods were obtained through experimental fatigue tests. A new fatigue life model was established and the *P*-*S*-*N* curves of two types of ultra-high strength sucker rods were obtained. For HL- and HY-type ultra-high strength sucker rods, the average error between the fitting result and fatigue test value is 1.25% and 4.39%, respectively. Compared to the *S*-*N* curve fitting result obtained from the Basquin model commonly used for sucker rods, the new model based on three-parameter Weibull distribution provides better fitting precision and can estimate fatigue limit more accurately, so this model is more suitable for estimating fatigue life and can better guide the design of ultra-high strength sucker rod strings.

## 1. Introduction

In recent years, with the continuous development of the oil production industry, class C and class D sucker rods have become incapable of meeting the production process requirements of special wells such as deep wells, ultra-deep wells, and heavy oil wells. Thus, ultra-high strength sucker rods are increasingly being used in oil fields because their strength and mechanical properties are better than those of class C and class D rods. It has been proved that sucker rod failure is mostly caused by fatigue or corrosion fatigue, so it is very important to study the fatigue problem of ultra-high strength sucker rod in rod design and safety evaluation. Specifically, it is crucial to study the fatigue performance of ultra-high strength sucker rods to obtain a reliable fatigue life prediction model through the laboratory experiments [1,2,3,4].

Sucker rods are subjected to cyclic alternating loads in complex working environments and fatigue fracture occurs after a certain number of cycles. The occurrence of fracture leads to a series of problems such as a reduction in oil recovery rate and production, as well as an increase in operation cost, which impacts the economic benefit of oil industries. For each sucker rod fracture accident, the equivalent loss can reach USD 3143-4715. Therefore, several methods have been proposed to prevent the fatigue damage of sucker rods. The fatigue life of sucker rods can be improved by a heat treatment process and by using new rod materials [5,6,7,8]. However, fatigue fracture is still unavoidable, so the study of the fatigue life of ultra-high strength sucker rods is of great significance to determine the performance of sucker rod strings and prevent premature fracture. Several researchers have examined the fatigue life of ultra-high strength sucker rods. For example, Lin Yuanhua et al. used the Forman model to predict the fatigue life of a sucker rod with or without initial crack [9]. Fan Song et al. assumed that sucker rod fatigue data obeyed normal distribution and derived a fatigue life prediction model by fitting the Basquin formula in the form of power function [10]. Li Qi et al. used the static characteristics of sucker rods to predict fatigue performance and proposed a fatigue life prediction method considering the cumulative damage factors [11]. Song Kaili et al. processed sucker rod fatigue data with log-normal distribution and obtained the maximum stress vs. fatigue life (*S*-*N*) curve in linear form [12]. Ding Wen et al. established a damage evolution model of a HL class sucker rod based on damage mechanics and simulated the fatigue life of this sucker rod under different damage forms by using the effective stress method and ANSYS software [13]. Most of the existing fatigue models for sucker rods are based on the Basquin linear model, which assumes that the *S*-*N* data obeys normal distribution. Using the least squares method to estimate the value of parameters *A* and *B*, the relationship between stress and high cycle fatigue life can be obtained, which is used to determine the *P*-*S*-*N* curve (where *P* is the reliability). However, the Basquin model has some limitations. Firstly, the model assumes a linear relationship between the maximum stress and the number of fatigue cycles, and the *S*-*N* curve can neither be extrapolated from the finite life region to the high-cycle fatigue life region, nor can it be used to estimate the fatigue limit of materials [14,15,16,17]. In addition, the model can only be used for estimation within the experimental range. Beyond the experimental range, the confidence of the model is greatly reduced, which can lead to erroneous results on the fatigue performance of ultra-high strength sucker rods. Therefore, it is necessary to establish a more reliable model to describe the *S*-*N* curve of sucker rods.

It is well known that the Weibull distribution is the most suitable distribution for survival and life analysis. Since it can easily calculate the distribution parameters by probability value, it is widely used in the processing of experimental data related to fatigue life [18,19,20,21]. In this study, the fatigue data of two kinds of ultra-high strength sucker rods (HL and HY class) was experimentally obtained. Based on the experimental data, a new three-parameter Weibull distribution model was established to study the fatigue performance of the sucker rod. The superiority of the proposed model was verified by comparing the fitting results with those obtained by the Basquin model based on normal distribution.

## 2. Fatigue Experiment

### 2.1. Experimental Equipment and Samples

**Figure 1:**
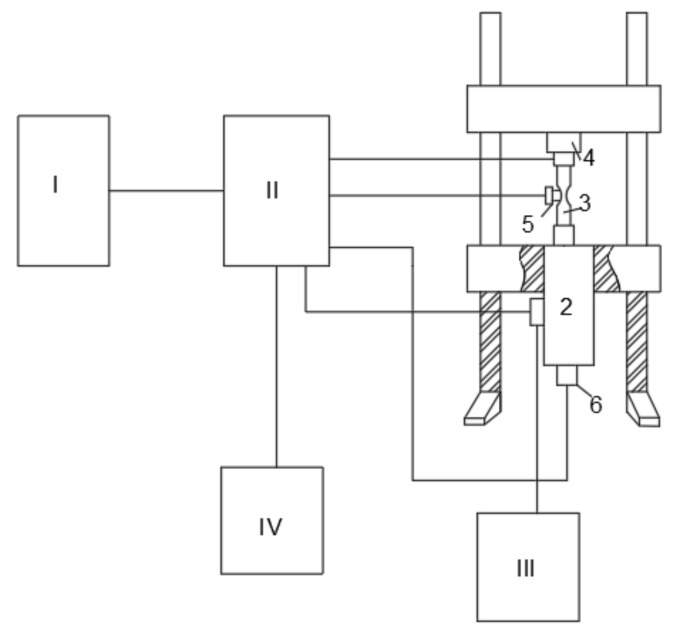
Schematic of the PLG-300C electromagnetic resonance high-frequency fatigue testing machine.

The commonly used fatigue testing machines include mechanical transmission, hydraulic transmission, electromagnetic resonance, and electro-hydraulic servo machine. In this study, an electromagnetic resonance high-frequency fatigue testing machine (PLG-300C Changchun Chuangyuan Test Equipment Co., LTD, Ji’nan, China) was used. Figure 1 shows a schematic of this fatigue testing machine. The signal through the servo controller I is sent to the servo valve 1 as the control signal, which is used to control the movement of high-pressure oil from high pressure hydraulic source III into actuator 2. The strain sensor and displacement sensor are used for the measurement of the sample in 3. Furthermore, the force, strain, and displacement are converted into electrical signals, which are used as the feedback to the servo controller with a given reference signal. The difference signal is sent to the servo valve to adjust the position of the actuator, and the process is repeated several times. Finally, the force (strain, displacement) on the sample reaches the required accuracy, and the other force, strain, and displacement signals are recorded in the reader unit IV.

**Figure 2:**
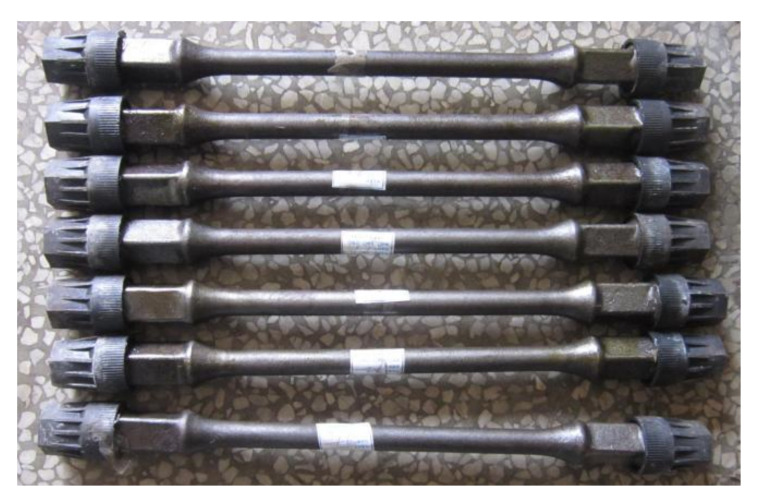
Actual images of sucker rod sample.

The sample sizes and specifications were considered in strict accordance with the oil and gas industry standard SY/T5029-2013. The samples were short sucker rods, mainly HL- and HY-type, with diameters of 19 mm and 22 mm, respectively, and lengths of 500 ± 50 mm. The production was consistent with the batch production process. Figure 2 shows the actual snapshots of these samples.

### 2.2. Experimental Steps

(1) The test specimen is prepared, and its original size is measured with a vernier caliper. The specimens with machining defects on their surfaces cannot be used.
(2) The testing machine is turned on, and various test parameters are set.
(3) The specimen is installed such that it is approximately coaxial with the test machine spindle.
(4) A certain number of specimens are tested. The test stress is not less than 500 MPa for pull-pull load with sinusoidal loading frequency below 150 Hz, and load ratio R = 0.1. The number of cycles *N* is observed, and the location of the fracture is recorded. The remaining specimens are tested under the same parameters.
(5) The specimen is removed after the test. The test site is cleaned, and the testing machine is restored.
(6) The relevant calculations are performed according to the test records.

### 2.3. Experimental Results

Table 1 and Table 2 show the fatigue test results of HL- and HY-type sucker rods, respectively, under different stresses. According to the experimental results, there is no obvious relationship between the fatigue fracture position of sucker rod and the stress and frequency.

**Figure 3:**
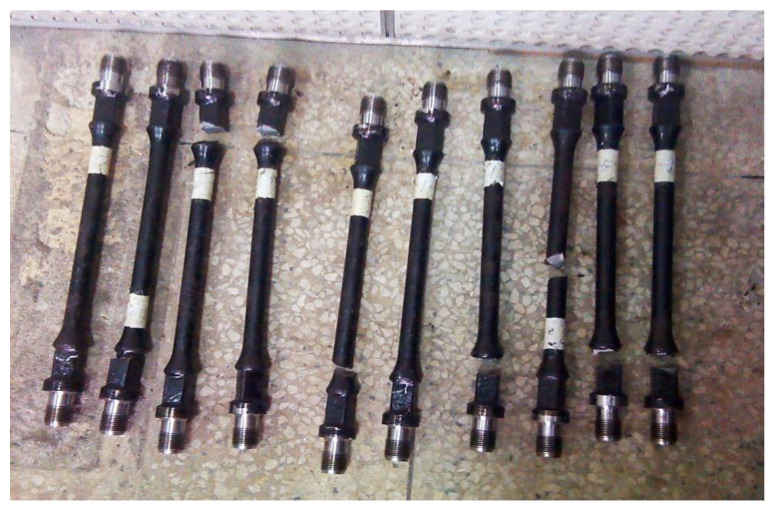
Some specimens after fatigue fracture.

The fatigue failure of sucker rod is due to its surface defects, resulting in stress concentration, which leads to crack formation, expansion, and fracture. The fracture locations observed in the test are summarized in Table 3. The main fracture location cannot be determined, and it is necessary to improve the surface finishing quality of the rod. The snapshots of some specimens after fracture are shown in Figure 3.

## 3. Fatigue Life Model Establishment

### 3.1. Fatigue Life Model of Sucker Rod Based on Weibull Distribution

The cumulative failure distribution function of Weibull distribution is [22]

(1) $$p\left(x\right)=1-\exp\left[-{\left(\frac{x-\alpha }{\beta }\right)}^{\gamma }\right]$$

where *x* is an independent variable (number of failure cycles or a function of number of failure cycles), α∈R is the position parameter (minimum life), β>0 is the scale parameter (characteristic life), and γ>0 is the shape parameter(slope of the cumulative distribution function).

The fatigue stress-life (*S*-*N*) function can be expressed as follows [23,24]:

(2) $$\log\left(\frac{S}{S_{0}}\right)\log\left(\frac{N}{N_{0}}\right)=D$$

where *S* is the maximum stress, *N* is the number of fatigue cycles (life), *S*_0_ is the maximum stress correction parameter, *N*_0_ is the life correction parameter, and *D* is a constant.

If the fatigue life of sucker rod obeys the three-parameter Weibull distribution: $X=\left(\log N-A\right)\left(\log S-B\right)$, then the fatigue stress-life (*S*-*N*) function model based on this distribution is

(3) $$p\left(\left.N\right|S\right)=1-\exp\left\{\left.{\left[\frac{\left(\log N-A\right)\left(\log S-B\right)-\alpha }{\beta }\right]}^{\gamma }\right\}\right.$$

where $\left(\log N-A\right)\left(\log S-B\right)\geq \alpha$. Here, *A* and *B* are the correction parameters.

According to Equation (1), the sucker rod fatigue test results were fitted to estimate the parameters *A*, *B*, and *α*, *β*, *γ*.

#### 3.1.1. Estimates of Parameters A and B

The constant *B* is replaced by the average value of the number of fatigue cycles (μ), and Equation (2) can be rewritten as follows:

(4) $$\log N=A+\frac{\mu }{\log S-B}$$

The linear regression method is used to estimate the values of *A* and *B*, and the minimum value of Equation (5) is required,

(5) $$Q=\sum_{i=1}^{n}{\left(\log N_{i}-A-\frac{\mu }{\log S_{i}-B}\right)}^{2}$$

where *n* is the sample size, and $N_{i}$ is the fatigue failure cycle number corresponding to each maximum stress $S_{i}$.

Based on fatigue data, the values of *A* and *B* can be estimated by using linear regression method according to Equations (4) and (5).

#### 3.1.2. Estimates of Parameters *α*, *β* and *γ*

When the values of parameters *A* and *B* are obtained, the probabilistic weighted moment method [25,26,27] can be used to estimate the values of α,β, and γ. First, the probabilistic weighted moment function of the three-parameter Weibull distribution can be written as follows:

(6) $$M_{1,0,s}=\frac{\alpha }{s+1}+\frac{\beta }{{\left(s+1\right)}^{1+\frac{1}{\gamma }}}\Gamma \left(1+\frac{1}{\gamma }\right)\begin{array}{cc} & \gamma >0 \end{array}$$

To estimate the parameters of the three-parameter Weibull distribution, three equations are needed. Therefore, if *s* = 0, 1, and 2 are substituted into Equation (6), the following equations can be obtained:

(7) $$\left\{\begin{array}{l} M_{1,0,0}=\alpha +\beta \cdot \Gamma \left(1+\frac{1}{\gamma }\right)\\ M_{1,0,1}=\frac{\alpha }{2}+\frac{\beta }{2^{1+\frac{1}{\gamma }}}\cdot \Gamma \left(1+\frac{1}{\gamma }\right)\\ M_{1,0,2}=\frac{\alpha }{3}+\frac{\beta }{3^{1+\frac{1}{\gamma }}}\cdot \Gamma \left(1+\frac{1}{\gamma }\right) \end{array}\right.$$

According to Equation (7), the three parameters of Weibull distribution can be expressed as follows:

(8) $$\alpha =M_{1,0,0}-\beta \cdot \Gamma \left(1+\frac{1}{\gamma }\right)$$

(9) $$\beta =\frac{2M_{1,0,1}-M_{1,0,0}}{\left(2^{\frac{-1}{\gamma }}-1\right)\Gamma \left(1+\frac{1}{\gamma }\right)}$$

(10) $$\frac{3M_{1,0,2}-M_{1,0,0}}{2M_{1,0,1}-M_{1,0,0}}=\frac{3^{\frac{-1}{\gamma }}-1}{2^{\frac{-1}{\gamma }}-1}$$

Then, according to the experimental data, the value of the probabilistic weighted moment $M_{1,0,s}$ can be obtained, and the three parameters α,β,γ are determined by using the Equations (8)–(10). The values of $M_{1,0,0}$, $M_{1,0,1}$ and $M_{1,0,2}$ can be calculated as follows:

(11) $${\hat{M}}_{1,0,0}=\frac{1}{n}\sum_{i=1}^{n}x_{i}$$

(12) $${\hat{M}}_{1,0,1}=\frac{1}{n\left(n-1\right)}\sum_{i=1}^{n}\left(n-i\right)x_{i}$$

(13) $${\hat{M}}_{1,0,2}=\frac{1}{n\left(n-1\right)\left(n-2\right)}\sum_{i=1}^{n}\left(n-i\right)\left(n-i-1\right)x_{i}$$

After calculating the five parameters of Weibull distribution model, the fatigue life functions of HL and HY sucker rods can be obtained.

### 3.2. Fatigue Life Model Based on Normal Distribution Basquin Formula

Presently, Basquin formula is applied for the *S*-*N* curve fitting of sucker rod. The Basquin formula mainly has two forms: exponential form and logarithmic form, which are expressed as follows [28]:

(14) $$S=C\cdot N^{m}$$

(15) logS=logC+mlogN

where *S* is the maximum stress, *N* is the fatigue life (number of failure cycles), and *C* and *m* are the parameters related to the loading mode of experimental materials.

It is assumed that logarithmic fatigue life $\log N_{p}$ follows normal distribution. In order to fit the *P-S-N* curve, it is necessary to obtain the fatigue life expression following the standard normal distribution. According to the normal distribution theory, the fatigue life following the standard normal distribution under the specified reliability is:

(16) $$\log N_{p}=\mu +\mu_{p}\sigma$$

In this formula, $\log N_{p}$ is the logarithmic fatigue life obeying the standard normal distribution; μ is the average logarithmic fatigue life; $\mu_{p}$ is the standard normal skewness corresponding to the reliability *P* (obtained by checking the standard normal skewness); σ is standard deviation of logarithmic fatigue life.

The least square method is used to fit the *P-S-N* curve, so that B=logC and A=m build the fitting equation: the formula is obtained in Equation (17):

(17) $$\log S=A\log N_{p}+B$$

(18) $$\left\{\begin{array}{l} \hat{A}=\frac{n\sum \log N_{pi}\log S_{i}-\sum \log N_{pi}\sum \log S_{i}}{n\sum {\left(\log S_{i}\right)}^{2}-{\left(\sum \log S_{i}\right)}^{2}}\\ \hat{B}=\frac{1}{n}\sum_{i=1}^{n}\log N_{pi}-\hat{A}\frac{1}{n}\sum_{i=1}^{n}\log S_{i} \end{array}\right.$$

According to the experimental fatigue data, *A* and *B* are obtained, and then the geometric parameters *m* and *C* of HY and HL sucker rods under different reliability values are obtained. Finally, the fatigue life function expression of sucker rod is obtained.

## 4. Regression Validation

### 4.1. Fatigue Life Model Validation of an HY-Type Ultra-High Strength Sucker Rod

#### 4.1.1. *P-S-N* Curve Fitting

According to the experimental data and the proposed three-parameter Weibull distribution model for parameter estimation, the *P*-*S*-*N* curve of an HY-type ultra-high strength sucker rod is fitted. The geometric parameters of the *P*-*S*-*N* curve are calculated as follows: A=−20.421,B=−0.9675,α=96.5449,β=0.6798, and γ=1.7588.

Now, for an HY-type ultra-high strength sucker rod, the *P*-*S*-*N* curve of the three-parameter Weibull distribution model considering different failure probabilities can be expressed as follows:

(19) $$p\left(\left.N\right|S\right)=1-\exp\left\{\left.{\left[\frac{\left(\log N+20.421\right)\left(\log S+0.9675\right)-96.5449}{0.6798}\right]}^{1.7588}\right\}\right.$$

**Figure 4:**
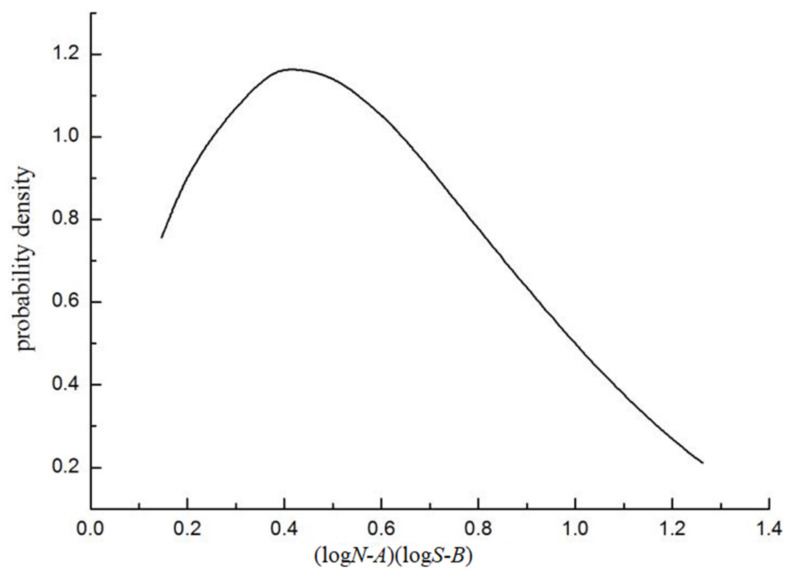
Probability density function of HY-type sucker rod.

According to the estimated values of position, scale, and shape parameters, the *P*-*S*-*N* curve expression is obtained, and the probability density function of Weibull distribution of HY-type sucker rod is determined, as shown in Figure 4.

It can be seen from the figure that the extreme value of probability density of HY-type sucker rod is located near 0.44, and after conversion, it is found that the fatigue life of a sucker rod is concentrated near 10^6^, which meets the requirements of practical application.

The experimental data were processed according to the Basquin model based on normal distribution, and the *S*-*N* curve geometric parameters of an HY-type ultra-high strength sucker rod under different failure probabilities were calculated. The results are shown in Table 4.

The *S*-*N* curve expressions of the normal distribution model with 99.99% and 50% reliability can be obtained as follows:

(20) logS=3.2653−0.096logN

(21) logS=3.5205−0.1373logN

#### 4.1.2. Results and Discussion

**Figure 5:**
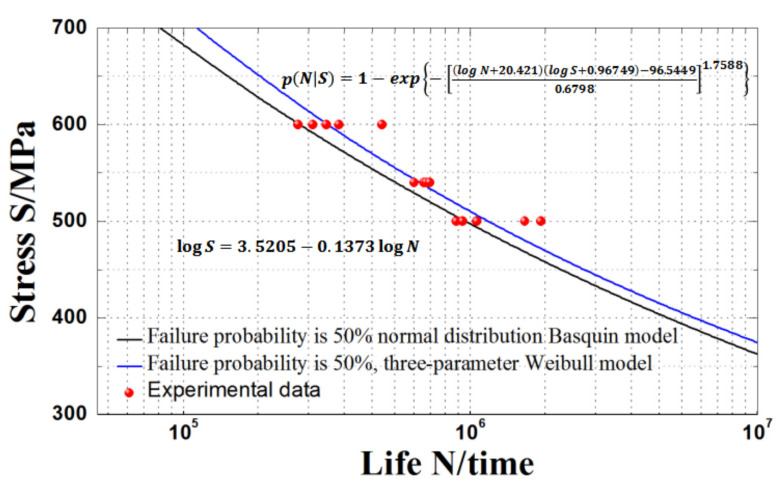
Comparison of *S*-*N* curve fitting results of HY-type ultra-high strength sucker rod obtained from the two models.

The curve fitting results based on the three-parameter Weibull distribution are compared with those based on the normal distribution Basquin model in Table 5 and Figure 5. It is clear that the average absolute error of the pre-experimental fatigue life based on *P*-*S*-*N* curve fitting of three-parameter Weibull distribution model and normal distribution Basquin model are 4.39% and 22.44%, respectively.

It is evident from Table 5 that when the maximum stress is 540 MPa, the error of the number of fatigue cycles obtained by the three-parameter Weibull distribution model is at least 3.35%, while that obtained by the normal distribution Basquin model is 20.27%, and it becomes 22.48% when the maximum stress is 500 MPa. Furthermore, when the fatigue life is higher, the fitting accuracy of the Basquin model based on normal distribution is lower, while that of the Basquin model based on three-parameter Weibull distribution is higher and the fitting error fluctuation is small. Therefore, it is reasonable to use the three-parameter Weibull distribution model to examine the fatigue life of an HY-type ultra-high strength sucker rod.

**Figure 6:**
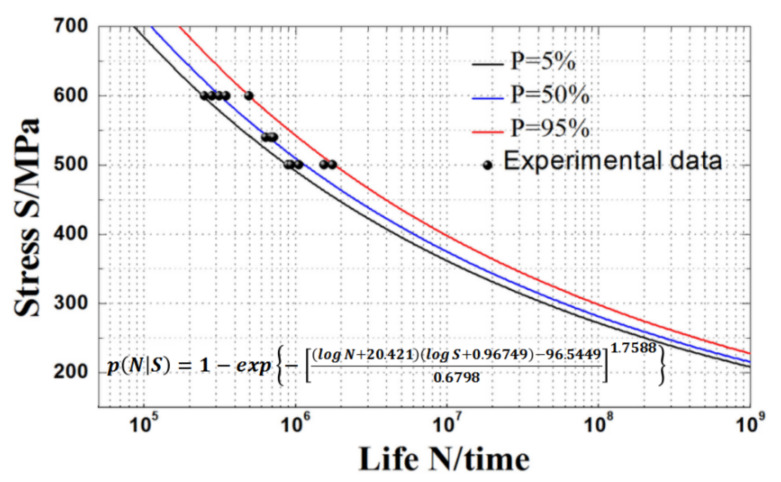
Fatigue *S*-*N* curve of HY-type ultra-high strength sucker rod obtained by three-parameter Weibull distribution model under different failure probabilities.

**Figure 7:**
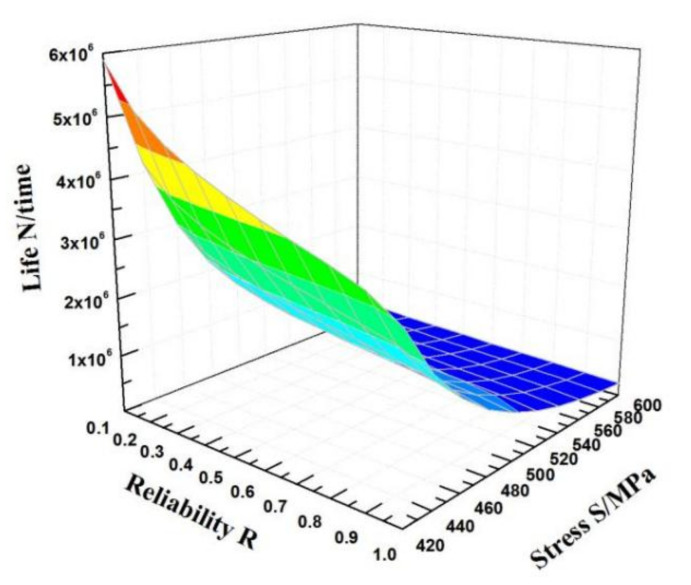
Variation in the fatigue life of HY-type sucker rod a function of stress and reliability.

According to Equation (19), the obtained *S*-*N* curves of an HY-type ultra-high strength sucker rod at failure probabilities of 5%, 50%, and 95% are shown in Figure 6. The fatigue test data are all between the curves with failure probabilities of 5% and 95%, and the curves obtained by fitting the fatigue life model based on three-parameter Weibull distribution has a good curvature and are more consistent with the experimental data. As the maximum stress and failure probability decrease, the curves obtained by fitting the fatigue life model based on three-parameter Weibull distribution gradually become gentle and approach the fatigue limit value, and the low curve dispersion under all probabilities is more consistent with the actual situation. Therefore, the *P*-*S*-*N* curve of the three-parameter Weibull model can effectively fit the fatigue performance of an HY-type ultra-high strength sucker rod under different failure probabilities. Figure 7 shows the variation in the fatigue *P*-*S*-*N* surface of an HY-type ultra-high strength sucker rod. It can be seen that the *P*-*S*-*N* curve is closer to *z*-axis when the maximum stress decreases and the reliability increases, and the fatigue life or fatigue strength calculated by *P*-*S*-*N* curve formula is more conservative.

### 4.2. Fatigue Life Model Validation of an HL-Type Ultra-High Strength Sucker Rod

#### 4.2.1. *P-S-N* Curve Fitting

According to the experimental data and the proposed three-parameter Weibull distribution model for parameter estimation, the *P*-*S*-*N* curve of an HL-type ultra-high strength sucker rod is fitted. The geometric parameters of *P*-*S*-*N* curve of HL-type sucker rod are calculated as A=3.8963,B=2.5152,α=0.3451,β=0.0334, and γ=2.6948.

Now, for the HL-type ultra-high strength sucker rod, the *P*-*S*-*N* curve of the three-parameter Weibull distribution model considering different failure probabilities can be expressed as follows:

(22) $$p\left(\left.N\right|S\right)=1-\exp\left\{\left.{\left[\frac{\left(\log N-3.8963\right)\left(\log S-2.5152\right)-0.3451}{0.0334}\right]}^{2.6948}\right\}\right.$$

The experimental data were processed according to the parameter estimation method of the Basquin model, based on normal distribution, and the *S*-*N* curve geometric parameters of an HL-type ultra-high strength sucker rod under different failure probabilities were calculated. The results are shown in Table 6.

The *S*-*N* curve expressions of the normal distribution model with 99.99% and 50% reliability can be obtained as follows:

(23) logS=3.2377−0.0914logN

(24) logS=3.4609−0.1287logN

#### 4.2.2. Results and Discussion

**Figure 8:**
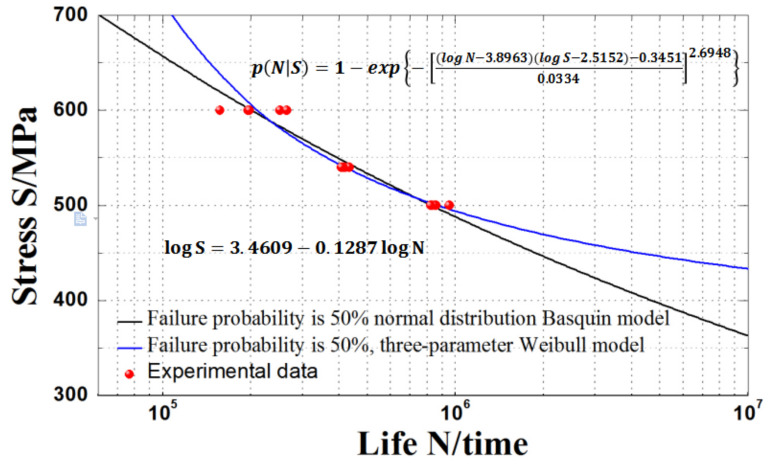
Comparison of *S*-*N* curve fitting results of HL-type ultra-high strength sucker rod obtained from the two models.

The curve fitting results based on the three-parameter Weibull distribution model are compared with those based on the normal distribution Basquin model in Table 7 and Figure 8. It can be seen from the table that the average absolute error of the pre-experimental fatigue life based on the *P*-*S*-*N* curve fitting of three-parameter Weibull distribution model and normal distribution Basquin model are 1.25% and 6.26%, respectively.

It is clear from Table 7 that when the maximum stress is 540 MPa, the error of the number of fatigue cycles obtained by the three-parameter Weibull distribution model is at least 0.63%, while that obtained by the normal distribution model is, at most, 8.74%. Furthermore, when the fatigue life is higher, the fitting accuracy of the Basquin model based on normal distribution is lower, while that of the Basquin model based on three-parameter Weibull distribution is higher and the fitting error fluctuation accuracy is small. Therefore, it is reasonable to use the three-parameter Weibull distribution model to examine the fatigue life of an HL-type ultra-high strength sucker rod.

**Figure 9:**
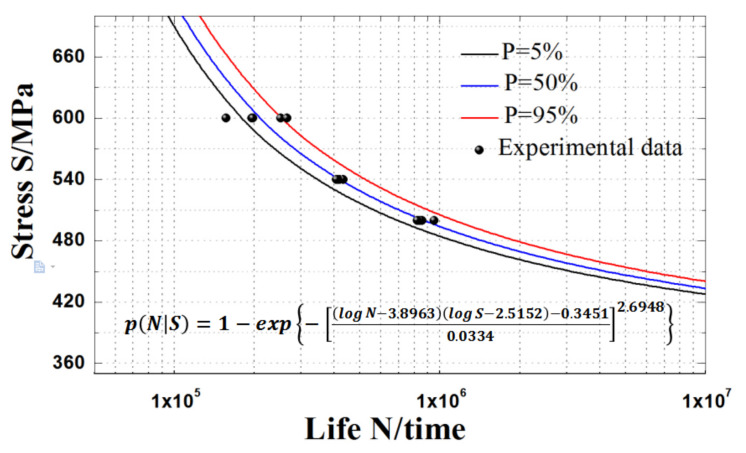
Fatigue *S*-*N* curve of HL-type ultra-high strength sucker rod obtained by the three-parameter Weibull distribution model under different failure probabilities.

**Figure 10:**
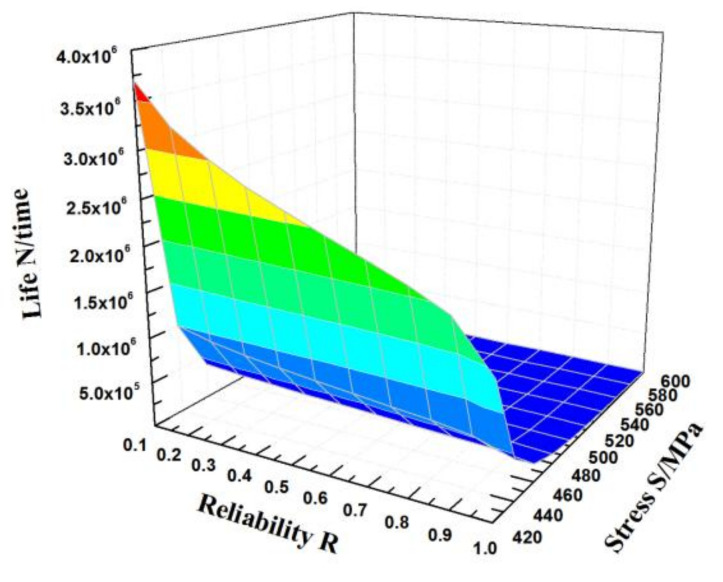
Variation in the fatigue life of HL-type sucker rod as a function of stress and reliability.

According to Equation (22), the obtained *S*-*N* curves of an HL-type ultra-high strength sucker rods at failure probabilities of 5%, 50%, and 95% are shown in Figure 9. The fatigue test data are all between the curves with failure probabilities of 5% and 95%, and the curves obtained by fitting the fatigue life model based on three-parameter Weibull distribution has a good curvature and are more consistent with the experimental data. As the maximum stress and failure probability decrease, the curves obtained by fitting the fatigue life model based on three-parameter Weibull distribution gradually becomes gentle and approaches the fatigue limit value, and the low curve dispersion under all probabilities is more consistent with the actual situation. Therefore, the *P*-*S*-*N* curve of the three-parameter Weibull model can well fit the fatigue performance of an HL-type ultra-high strength sucker rod under different failure probabilities. Figure 10 shows the fatigue *P*-*S*-*N* surface variation of an HL-type ultra-high strength sucker rod. It can be seen that when the maximum stress decreases and the reliability increases, the *P*-*S*-*N* curve becomes closer to the *z*-axis, and the fatigue life value or fatigue strength calculated by the *P*-*S*-*N* curve expression is more conservative.

By comparing the fitting results obtained by the proposed model based on three-parameter Weibull distribution and the common Basquin model based on normal distribution, it is clear that the proposed model is more suitable for data processing. For the proposed model, the *P*-*S*-*N* curve shape of a sucker rod more consistent with the actual situation, and the fitting precision is higher. Based on this model, the fatigue life of an ultra-high strength sucker rod under specific failure probability can be predicted by the curve equation under different reliabilities, which can provide a basis for the reliable design of an ultra-high strength sucker rod and the service life evaluation of rod string.

## 5. Conclusions

In this study, based on reasonable assumptions, the fatigue life models of two kinds of ultra-high strength sucker rods were established using three-parameter Weibull distribution. According to the fitting results, it was found that the proposed model could be used to effectively predict the fatigue life of ultra-high strength sucker rods with specific failure probability, which is potentially useful to evaluate the residual life and fatigue reliability of in-service sucker rods in the oil production field.

The fatigue experiments of two types of ultra-high strength sucker rods were conducted, and the experimental data were processed by normal distribution and Weibull distribution. The two fatigue life prediction models of ultra-high strength sucker rods were fitted and compared.

Weibull distribution was used to process the sucker rod fatigue data, and a new sucker rod fatigue life prediction model was established. The parameters of the model were estimated by the least squares method and probabilistic weighted moment method, and the *S*-*N* curve of an ultra-high strength sucker rod under different failure probabilities was obtained.

According to the fitting results of the two models, the average error between the number of fatigue failure cycles of an HY-type and HL-type sucker rod based on the three-parameter Weibull distribution model and the experimental value was 4.39% and 1.25%, respectively, which was far lower than the values of 19.84% and 12.50% obtained by the common Basquin model based on the normal distribution. Therefore, the proposed three-parameter Weibull distribution has better fitting accuracy and is more suitable for describing the fatigue life of ultra-high strength sucker rods.

## Figures and Tables

**Table 1:** Fatigue test results of HL-type sucker rods.

Diameter (mm)	Maximum Stress (MPa)	Maximum Load (kN)	Minimum Load (kN)	Stress Ratio	Life *N* (Cycles)	Fracture Location
22.23	500	193.96	19.40	0.1	833,120	Stress groove
22.32		195.54	19.55		767,320	Wrench party
22.38		196.59	19.66		853,620	Wrench party
22.37		196.41	19.64		722,682	Wrench party
22.32		195.54	19.55		753,018	Stress groove
19.19	540	173.45	17.35		420,356	Screw thread
19.18		173.27	17.33		419,652	Screw thread
19.11		172.01	17.2		433,111	Screw thread
19.20		173.63	17.36		413,256	Screw thread
19.09		171.65	17.17		408,352	Screw thread
18.95	600	169.14	16.91		195,968	Screw thread
19.18		173.27	17.33		251,597	Wrench party
18.85		167.36	16.74		265,439	Screw thread
19.02		170.39	17.04		197,851	Wrench party
19.08		171.47	17.15		156,850	Screw thread

**Table 2:** Fatigue test results of HY-type sucker rods.

Diameter (mm)	Maximum Stress (MPa)	Maximum Load (kN)	Minimum Load (kN)	Stress Ratio	Life *N* (Cycles)	Fracture Location
22.24	500	194.14	19.41	0.1	1,757,824	Rod body
22.10		191.70	19.17		1,545,593	Rod body
22.19		193.27	19.33		938,732	Wrench party
22.31		195.36	19.54		893,183	Rod body
22.20		193.44	19.34		1,053,226	Wrench party
18.94	540	152.06	15.21		635,986	Rod body
19.18		155.94	15.59		720,968	Rod body
19.40		159.54	15.95		701,872	Rod body
19.10		154.64	15.46		689,321	Rod body
19.28		157.57	15.76		687,367	Rod body
19.01	600	170.21	56.17		250,950	Wrench party
18.91		168.42	55.58		314,635	Rod body
19.02		170.39	56.23		347,449	Screw thread
18.90		168.25	55.52		281,548	Screw thread
19.17		173.09	57.12		491,519	Screw thread

**Table 3:** Fatigue fracture position statistics.

Serial Number	Fracture Location	Number	Ratio (%)
1	Rod body	9	16.6
2	Wrench party	23	42.5
3	Stress groove	7	13.0
4	Screw thread	14	26.0
5	Flange	1	1.9

**Table 4:** Estimated geometric parameters of HY-type sucker rod.

Reliability	99.99%		50%	
Geometric parameters	*A*	*B*	*A*	*B*
	−0.0960	3.2653	−0.1373	3.5205

**Table 5:** Comparison of fatigue life of HY-type ultra-high strength sucker rod based on the three-parameter Weibull distribution model and the normal distribution Basquin model.

Stress (MPa)	Experimentally Determined Life (Cycles)	Three-Parameter Weibull Distribution Model		Basquin Model Based on Normal Distribution	
		Life (Cycles)	Error (%)	Life (Cycles)	Error (%)
500	1237711	1,188,580	3.97%	959,431	22.48%
540	687102	664,063	3.35%	547,811	20.27%
600	337220	317,407	5.88%	254,353	24.57%

**Table 6:** Estimated geometric parameters of a HL-type sucker rod.

Reliability	99.99%		50%	
Geometric parameters	*A*	*B*	*A*	*B*
	−0.0914	3.2377	−0.1287	3.4609

**Table 7:** Comparison of fatigue life of HL-type ultra-high strength sucker rod based on the three-parameter Weibull distribution model and the normal distribution Basquin model.

Stress (MPa)	Experimentally Determined Life (Cycles)	Three-Parameter Weibull Distribution Model		Basquin Model of Normal Distribution	
		Life (Cycles)	Error (%)	Life (Cycles)	Error (%)
500	864,152	856,698	0.86%	828,252	4.15%
540	418,945	416,321	0.63%	455,548	8.74%
600	213,541	208,726	2.26%	200,956	5.89%

## Data Availability

Data available on request. The data presented in this study are available on request from the corresponding author.

## Nomenclature

*P*	failure probability
*S*	Maximum stress/MPa
*N*	number of fatigue cycles (life)
*x*	independent variable (number of failure cycles or a function of number of failure cycles)
*α*	position parameter (minimum life)
*β*	scale parameter (characteristic life)
*γ*	shape parameter (slope of the cumulative distribution function)
*S*_0_	maximum stress correction parameter/MPa
*N*_0_	life correction parameter
*A*	correction parameters
*B*	correction parameters
*n*	sample size
$N_{i}$	the fatigue failure cycle number corresponding to each maximum stress $S_{i}$
*S_i_*	Maximum stress
*C*	parameters related to the loading mode of experimental materials
*m*	parameters related to the loading mode of experimental materials
$\log N_{p}$	logarithmic fatigue life obeying the standard normal distribution
μ	average logarithmic fatigue life
$\mu_{p}$	standard normal skewness corresponding to the reliability P
σ	standard deviation of logarithmic fatigue life.
